# A Study on the Microstructure and Properties of Cu-Fe-Mg-Ti Alloys Based on Composition Regulation

**DOI:** 10.3390/ma18061325

**Published:** 2025-03-17

**Authors:** Yu Ding, Xiangpeng Xiao, Dawei Yuan, Jinshui Chen

**Affiliations:** 1 Advanced Copper Industry College, Jiangxi University of Science and Technology, Ganzhou 341000, China; 15755317102@163.com (Y.D.); yuan_dw@126.com (D.Y.); chenjinshui0797@163.com (J.C.); 2 Faculty of Materials Metallurgy and Chemistry, Jiangxi University of Science and Technology, Yingtan 335000, China

**Keywords:** Cu-Fe-Mg-Ti alloy, aging precipitation, pre-cooling deformation, heat resistance softening

## Abstract

This study systematically investigates how Fe-Ti atomic ratios (1:1, 1:2, and 2:1) influence the microstructure, mechanical properties, and softening resistance of Cu-Fe-Mg-Ti alloys under fixed total Fe + Ti content. Through hardness testing, electrical conductivity measurements, and multiscale characterization (optical microscopy, scanning/transmission electron microscopy, and X-ray diffraction), we reveal a previously unreported phenomenon: Ti-dominated ratios (1:2) enable superior strength–conductivity synergy. After 70% cold rolling and 550 °C aging, the alloy with a 2:1 Fe/Ti ratio exhibits peak hardness (166.5 HV) and conductivity (64.1% IACS), outperforming both 1:1 (173.9 HV, 51.3% IACS) and 1:2 (189.5 HV, 44.2% IACS) counterparts. Critical microstructure analysis confirms that increased Ti content promotes high-density Fe_2_Ti nanoprecipitation (5–15 nm) with coherent interfaces, enhancing strength while mitigating electron scattering. This work establishes atomic ratio optimization as a novel strategy to break the traditional strength–conductivity trade-off in copper alloys, providing a 21% hardness improvement over conventional Fe-Ti systems without sacrificing essential electrical performance.

## 1. Introduction

The two cornerstones of modern electronic information technology are integrated circuits and information displays. With the rapid development of electronic communications and other related information industries, the requirements for integrated circuits have gradually increased [1,2,3]. Leadframe materials are the main structural materials for IC (integrated circuit) packaging, and copper alloy materials are an important part of leadframe materials. In order to meet the requirements of the high-density assembly of integrated circuit products, integrated circuit leadframe materials with high density combined with high performance, high integration, and multi-lead and narrow pitch are being developed [4,5,6,7]. Leadframe materials currently available on the market include Cu-Fe-P alloys [8,9,10], Cu-Cr-Zr alloys [11,12,13], Cu-Ni-Si alloys [14,15,16], and other series of alloys [17,18,19].

Numerous studies have shown that the addition of Fe [20] and Ti [21] elements can effectively strengthen pure Cu in Cu-Fe and Cu-Ti binary alloys. α-Fe [22] and γ-Fe [23] nanoparticles can be precipitated from the Cu matrix during aging to strengthen the alloys. Shi et al. [24] prepared a Cu-1 wt.% Fe alloy and found that the alloy has higher tensile strength (TS) (194 MPa) than pure Cu (131 MPa). Binary Cu-Ti alloys also have high strength due to the spinodal decomposition and formation of β-Cu4Ti and β-Cu3Ti precipitates [25]. A TS of 760 MPa and an EC of 26.5% IACS were achieved in a Cu-1.5 wt.% Ti alloy [26]. Although Fe and Ti significantly enhance the mechanical strength of Cu alloys, they markedly degrade electrical conductivity (EC) [27,28]. This phenomenon arises because Fe and Ti exhibit the highest reduction rates in EC among alloying elements, combined with their relatively higher solid solubility in Cu compared to elements like Nb and Cr at low temperatures. Therefore, the lower EC limits the application of Cu-Fe and Cu-Ti alloys.

**Figure 1:**
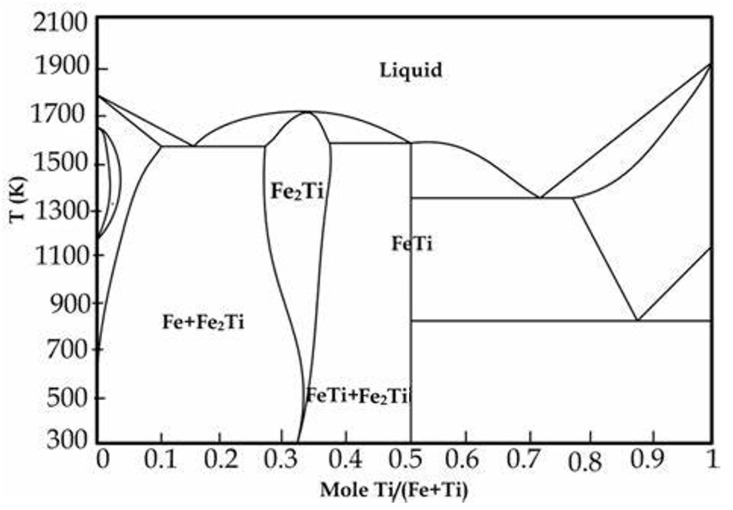
Fe-Ti Binary Alloy Phase Diagram.

Cu-Fe alloys are the mainstream alloys for leadframe materials and are widely used in the domestic market. The widely used copper alloys for leadframes are C19200 (Cu-0.1Fe-0.03P), C19400 (Cu-2.3Fe-0.1Zn-0.03P), and other alloys, although the C19200 alloy has an electrical conductivity of 90% IACS or more. However, its tensile strength is relatively low, limiting the scope of its application. In comparison, the C19400 alloy exhibits superior tensile strength (up to 450 MPa), with a conductivity of 60% IACS, although its softening temperature is only 475 °C, making it difficult to meet the needs of copper alloys for leadframes. According to Figure 1, the Fe-Ti phase diagram shows that iron (Fe) and titanium (Ti) elements can combine with each other to form iron-titanium (Fe-Ti) precipitated phases [29]. Preliminary studies have found that the tensile strength of Cu-0.74Fe-0.33Ti alloys can be as high as 590 MPa, and the electrical conductivity can be as high as that of C19400 (69% IACS), but scholars have not studied it thoroughly, and the alloy’s resistance to softening remains unclear. In this research, we explored the impact of the composition of Cu-Fe-Mg-Ti alloys with varying Fe/Ti atomic stoichiometry ratios on the mechanical and electrical properties of the alloys. We also examined the relationship between the alloys’ properties and their microstructure. Additionally, the alloys’ high-temperature resistance to softening and the softening mechanism were investigated.

## 2. Materials and Methods

The raw materials consisted of copper rods with a purity of 99.9 wt.%, a Cu-50 wt.% Ti intermediate alloy, pure Mg grains at 99.9 wt.%, pure Co blocks of 99.9 wt.%, and iron powder with a purity of 99.9 wt.%. The alloy was fabricated by melting it in a vacuum induction melting furnace under an argon atmosphere. Subsequently, casting was carried out using graphite molds, yielding a final ingot weight of approximately 2.3 kg. The designed composition of the alloy is presented in Table 1.

Hardness and electrical conductivity were measured using a micro-Vickers hardness tester (HUA YIN 200HVS-5) and a digital eddy current metal conductance tester (Sigma2008B/C). The hardness tester exerted a compressive load of 500 N (Newtons) on the specimen surface with a dwell time of 5 s during the standardized hardness measurement procedure. The samples were mechanically polished and then corroded with 10 g FeCl_3_ + 20 mL HCl + 300 mL H_2_O. The microstructure of the samples was investigated using an OM (optical microscope, ZEISS, Oberkochen, Germany) and emission SEM (scanning electron microscope, Tescan-Orsay Mira3, LMH) equipped with EDS (energy dispersive spectroscopy) and EBSD (electron back scattering diffraction). The samples for EBSD measurements were prepared by mechanical polishing with sandpaper followed by electropolishing using a solution of 30% phosphoric acid. The second phase of the alloys was observed and analyzed using a TEM (transmission electron microscope, TecnaiG2-20). We prepared the transmission samples by electropolishing them in 75% methanol and 25% nitric acid (volume fraction) at −30 °C.

The as-cast alloy was cropped, milled, and homogenized at 800 °C for 1 h in a box-type resistance furnace, followed by hot rolling with 60% reduction. Solution treatment was performed at 900 °C for the Cu-Fe-Mg-Ti alloy. Cold rolling with reductions of 30%, 50%, 70%, and 90% was then applied to the solution-treated samples. Aging treatments under varying temperatures and durations were systematically conducted to determine the optimal aging parameters through a comparative analysis of mechanical and electrical properties.

The optimal cold-rolled and aged alloys (5 mm × 7 mm specimens) were subjected to standardized anti-softening evaluation according to GB/T33370-2016. The specimens were heated at 20 °C increments (1 h dwell per temperature), with post-annealing hardness measurements defining the softening resistance curve. The critical anti-softening temperature was determined as the point where hardness reached 80% of its initial value (HV_initial_ = 189.5), establishing a quantitative threshold for thermal stability.

## 3. Results

### 3.1. Mechanical Properties

**Figure 2:**
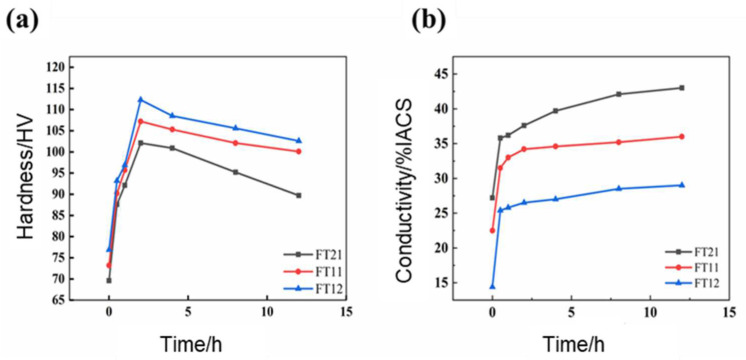
Hardness and electrical conductivity time evolution curves at 550 °C for three groups of alloys: FT12, FT11, and FT21. (**a**) Hardness curve. (**b**) Electrical conductivity curve.

Three groups of alloys were first solidified at 850 °C for a duration of 1 h and subsequently aged at 550 °C. The mechanical and electrical properties of the solid solution alloys throughout the ensuing aging treatment process are illustrated in Figure 2. As is evident from Figure 2a, the hardness curves for the two groups of alloys exhibited a rapid ascent to their peak values and then declined as the aging time was further extended. It is clear that both groups of alloys manifested an obvious aging strengthening effect. As can be observed from Figure 2b, the electrical conductivity curves for the two groups of alloys all reached a state of stabilization after initially surging rapidly to their peak values as the aging time increased.

During the aging process of the copper–iron–magnesium–titanium alloy, the initial increase in hardness arises from the precipitation of solute atoms, which obstruct dislocation motion and enhance strength. However, as aging progresses, precipitate coarsening reduces their effectiveness, leading to a decline in hardness. For conductivity, the rapid initial rise results from reduced electron scattering due to fine precipitates, but slower improvements occur as larger precipitates reintroduce scattering effects.

As shown in Figure 2, after aging for 4 h, the FT12 alloy attains its peak hardness, reaching 112.3 HV, with a conductivity of 27% IACS. For the FT11 alloy, peak hardness is achieved after 2 h of aging, with a value of 107.2 HV, along with a conductivity of 34.6% IACS. Similarly, the FT21 alloy reaches its hardness apex after 2 h of aging, with a hardness value of 102.1 HV and a conductivity of 34.6% IACS. However, it should be noted that there seems to be a discrepancy in the data provided for the FT21 alloy as it was found later that after 2 h of aging, it reaches a peak hardness of 107.2 HV and an electrical conductivity of 39.7% IACS.

Upon comparing the peak performances of these three groups of alloys, it becomes evident that when the iron-to-titanium ratio is 1:2, the alloy exhibits the highest peak hardness. In fact, it surpasses the hardness of the FT21 alloy by 10.2 HV. Nevertheless, this comes at a cost as the electrical conductivity of the FT12 alloy is significantly compromised, showing a reduction of 11.3% IACS compared to that of the FT21 alloy. Overall, it can be concluded that when the Fe-Ti ratio is 2:1, the alloy demonstrates a more favorable overall performance in comparison to other ratios.

**Figure 3:**
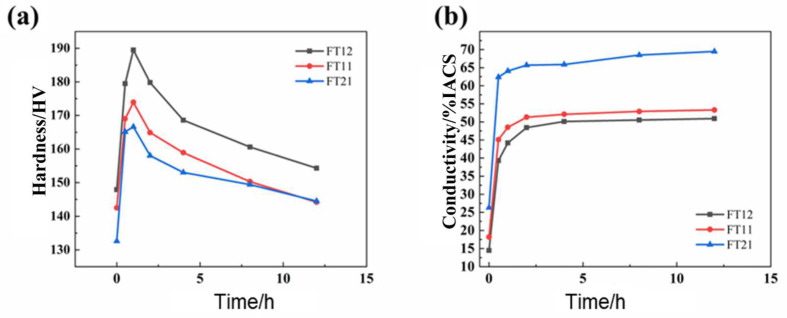
Conductivity versus hardness curves for three groups of alloys (FT12, FT11, and FT21), aged at 550 °C over time after 70% cold rolling. (**a**) Hardness curve. (**b**) Electrical conductivity curve.

The alloy was cold-rolled with a deformation of 70% and then aged at 550 °C. The mechanical and electrical properties of the alloy in the cold-rolled state during the subsequent aging treatment are shown in Figure 3. As can be seen in Figure 3a, the hardness curves for the alloys all increased rapidly to the peak and then decreased after the aging time was prolonged. The alloys have an obvious aging strengthening phenomenon. As seen in Figure 3b, the electrical conductivity curves for the alloys are all stabilized after a rapid increase to the peak value after extending the aging time.

As can be seen from Figure 3, the peak aging time of the FT12 alloy is 1 h, the peak hardness of the alloy is 189.5 HV, and the conductivity is 44.2% IACS; the peak aging time of the FT11 alloy is 1 h, the peak hardness of the alloy is 173.9 HV, and the conductivity is 51.3% IACS; the peak aging time of the FT21 alloy is 1 h, the peak hardness of the alloy is 166.5 HV, and the conductivity is 64.3% IACS. The peak hardness of the FT21 alloy is 166.5 HV and the electrical conductivity is 64.1% IACS.

### 3.2. Microstructure of the Alloy

**Figure 4:**
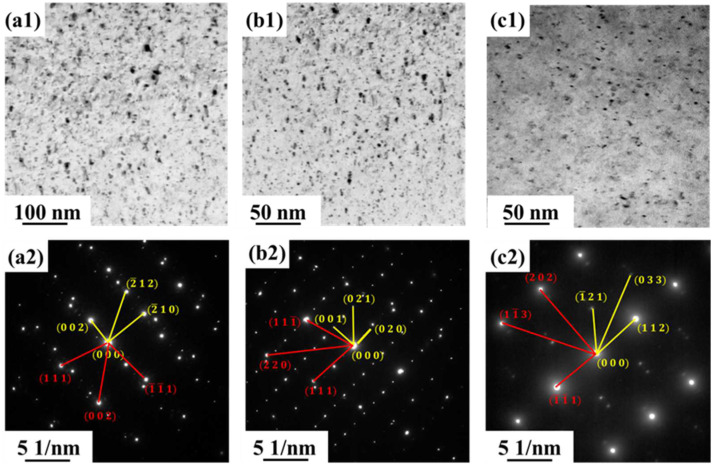
Bright-field TEM images of three groups of alloys in the peak aging state at 550 °C and their corresponding electron diffraction photographs: (**a1**,**a2**) FT12 alloy; (**b1**,**b2**) FT11 alloy; and (**c1**,**c2**) FT21 alloy.

**Figure 5:**
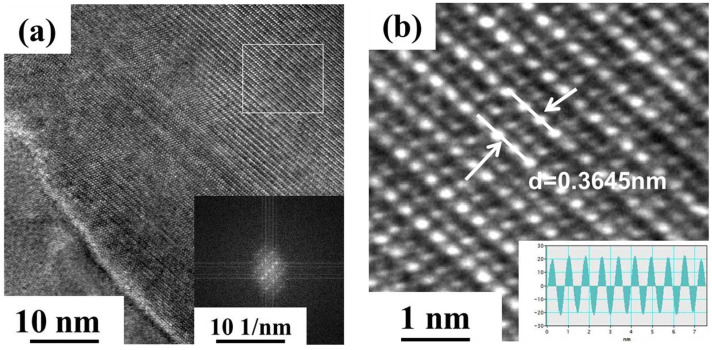
TEM images of the FT12 alloy supersaturated solid solution aged at 550 °C for 2 h: (**a**) HRTEM (High-Resolution Transmission Electron Microscopy) image and (**b**) corresponding IFFT (Inverse Fast Fourier Transform) image of (**a**).

Figure 4 shows the TEM images and corresponding selected area diffractograms (SADP) of peak-aged samples of three alloys (FT12, FT11, and FT21) at 550 °C, where Figure 4(a1,a2) show the FT12 alloy, Figure 4(b1,b2) show the FT11 alloy, and Figure 4(c1,c2) show the FT12 alloy; the yellow portion in the SADP is labeled as the precipitation phase, and the red part is the Cu phase. From Figure 4(a1,a2), it can be seen that the precipitated phase is in the form of particles and the diffraction spots of the corresponding precipitated particles appear clearly in the corresponding SADP. After the analysis of the electron diffraction pattern, it was found that the precipitated particles can be defined as the Fe_2_Ti phase, and the lattice constants of the hexagonal crystalline system of the Fe_2_Ti phase are a = 0.478 nm and c = 0.781 nm; there is also a [120]_Fe2Ti_//[$\bar{1}10$]_Cu_ site-directional relationship. From Figure 4(b1,b2), it can be seen that the precipitated phase of the FT11 alloy also shows a granular shape, which can be defined as the Fe_2_Ti phase, and there is a site-directional relationship of [100]_Fe2Ti_//[$\bar{1}10$]_Cu_ between the Fe_2_Ti phase and the matrix. Figure 4(c1,c2) show the peak aging precipitated phase state of the FT12 alloy; the precipitated particles can also be defined as the F_e2_Ti phase, and there is a site-directional relationship of [$\bar{1}\bar{1}1$]_Fe2Ti_//[$\bar{1}21$]_Cu_ between the Fe_2_Ti phase and the matrix. Figure 5 shows the HRTEM image of the precipitated phase of the FT12 alloy, which was transformed by FFT to reveal that the precipitated crystal structure is similar to the Fe_2_Ti phase, with a crystal plane spacing of 0.3645 nm.

### 3.3. High-Temperature Resistance to Softening

**Figure 6:**
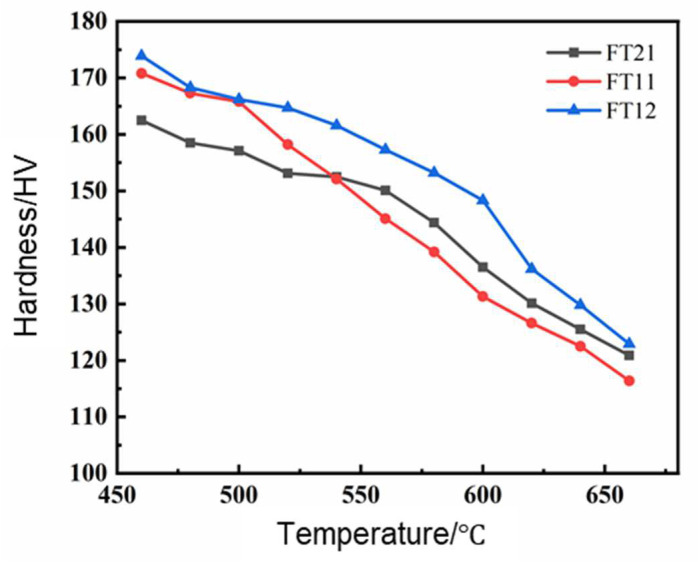
Softening property curves for peak-aged samples of FT21, FT11, and FT12 alloys after 70% cold rolling.

According to the aging performance change curve in Figure 3, the alloy was selected after cold rolling at 70% and aging at 550 °C for a high-temperature resistance to softening test at different temperatures of insulation for 1 h to measure the hardness of the alloy and draw its high-temperature resistance to softening curve. Figure 6 shows the high-temperature softening curves for the three groups of alloys.

Figure 6 shows that in the annealing temperature range of 460–500 °C, the hardness of the three groups of alloys decreases slowly; the hardness of the alloys in this annealing temperature range can reach more than 95% of the initial value, and the trend of a decrease in hardness is not significant. In the annealing temperature range of 520–660 °C, the hardness of the three groups of alloys decreases at an increasing rate, and the hardness values decrease significantly. The difference in the decreasing trend of alloy hardness between the two annealing temperature ranges indicates the existence of different softening mechanisms in the alloys at different annealing stages. The reason for the decrease in alloy hardness with the increase in holding temperature is that at lower holding temperatures, the diffusion power of atoms inside the alloy increases, the precipitated phase grows and coarsens, the ability to hinder dislocation movement decreases, and alloy hardness decreases, but due to the lower holding temperature, the over-aging reaction is not significant and the decrease in alloy hardness is not obvious. When the insulation temperature is too high, the alloy is subject to more serious over-aging; at this time, the decline in alloy hardness increases. So, in the 520–660 °C annealing temperature range, alloy hardness is significantly reduced. It can be seen from the figure that the hardness of the FT12 alloy is higher than the remaining two groups of alloys, and the FT11 alloy’s insulation temperature is lower when the hardness value is higher than that of FT21 alloy, but when the insulation temperature is more than 540 °C, alloy hardness decreases rapidly, the overall hardness curve decreases, and the FT21 alloy transitions to the FT11 alloy more gently. The hardness value of the alloy decreases to 80% of the initial value after 1 h of annealing treatment, at which time, the corresponding annealing temperature is the softening temperature of the alloy. It can be seen that the FT11 alloy has a softening temperature of 560 °C, the FT12 alloy has a softening temperature of 580 °C, and the FT21 alloy has a softening temperature of 600 °C. The FT21 alloy has a softening temperature of 580 °C. Compared to the FT11 alloy, the softening temperature of the FT21 alloy is increased by 40 °C and that of the FT12 alloy is increased by 20 °C, indicating that the alloy has the best ability to resist softening at high temperatures when the ratio of iron to titanium is 2:1.

**Figure 7:**
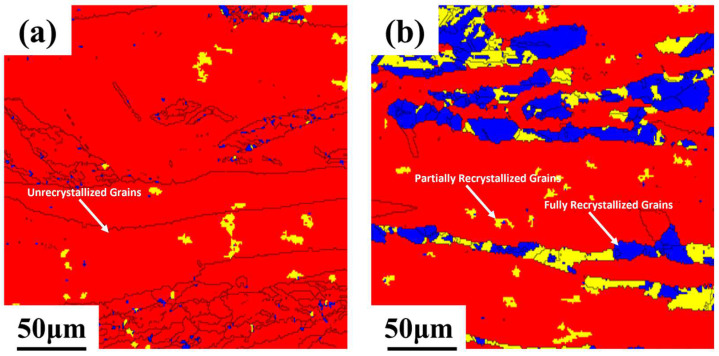
EBSD plots of FT21 alloy annealed for 1 h at different temperatures: (**a**) 500 °C and (**b**) 620 °C.

Figure 7 shows the EBSD plots of FT21 alloys after peak aging at 500 °C and 620 °C for 70% deformation. In Figure 7, the red regions correspond to unrecrystallized grains retaining deformed microstructures, the blue areas indicate partially recrystallized grains containing substructures, and the yellow zones denote fully recrystallized equiaxed grains. Table 2 shows the EBSD plots of Figure 7 with the percentage of recrystallization of the alloys. As shown in Table 2, the recrystallization percentage of the FT21 alloy is 2.8% after 500 °C × 1 h holding time, and the recrystallization percentage of the FT21 alloy held at 620 °C × 1 h is higher, reaching 28.6%.

**Figure 8:**
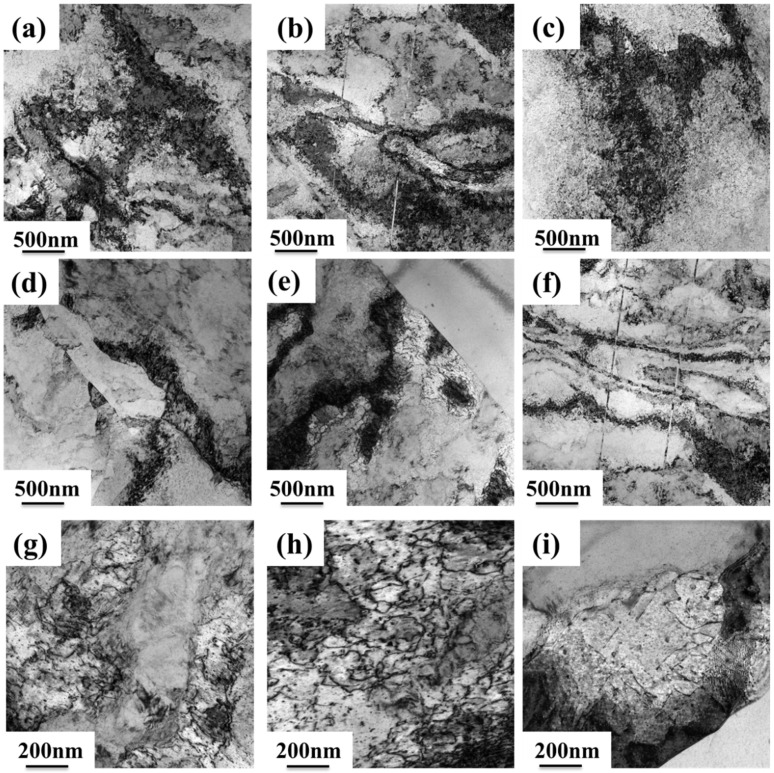
TEM images of peak-aged samples of three alloys (FT12, FT11, and FT21), held at different temperatures for 1 h after 70% cold rolling: (**a**,**d**,**g**) FT12 alloy; (**b**,**e**,**h**) FT11 alloy; and (**c**,**f**,**i**) FT21 alloy at (**a**–**c**) 500 °C; (**d**–**f**) 580 °C; and (**g**–**i**) 620 °C.

**Figure 9:**
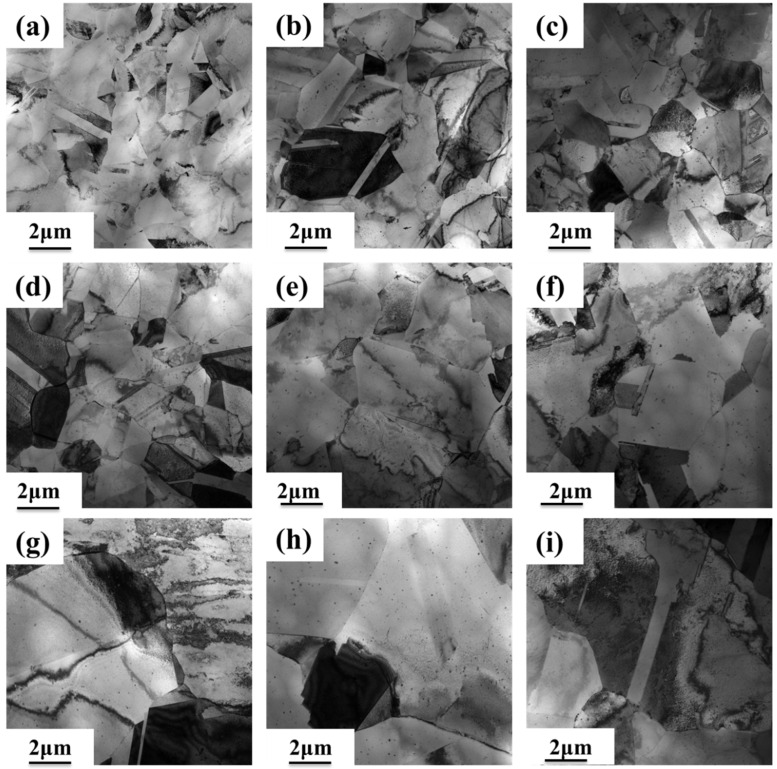
TEM images of peak-aged samples of three alloys (FT12, FT11, and FT21), held at different temperatures for 1 h after 70% cold rolling: (**a**,**d**,**g**) FT12 alloy; (**b**,**e**,**h**) FT11 alloy; and (**c**,**f**,**i**) FT21 alloy at (**a**–**c**) 500 °C; (**d**–**f**) 580 °C; and (**g**–**i**) 620 °C.

Figure 8 and Figure 9 show the TEM images of three groups of Cu-Fe-Mg-Ti alloys after peak aging samples were annealed at different temperatures for 1 h after 70% cold rolling. From Figure 8, it can be seen that after the three groups of alloys were insulated at 500 °C × 1 h, there were high-density dislocations and dislocation entanglement inside the alloys. After the three groups of alloys received 580 °C × 1 h heat preservation treatment, the density of dislocations in the alloy was smaller than that of the alloy after 500 °C × 1 h heat preservation treatment. This is due to high-temperature annealing; the alloy appears to be affected by recrystallization and dislocation movement, the same slip surface of the dislocations of the same number of dislocations are attracted to each other, and dislocation arrangement gradually leads to regularization. When the three groups of alloys are annealed at 620 °C for 1 h, the dislocation density decreases dramatically, the phenomenon of mutual entanglement between dislocations and the dislocation cell basically disappears, the dislocations in the cell wall are gradually transformed into the dislocation network in the low-energy state, and there is a thin and long dislocation line. The recrystallized grains of the three groups of alloys after annealing at 500 °C for 1 h can be clearly observed in Figure 9, and when the alloy is annealed at 580 °C for 1 h, the degree of recrystallization is intensified, and the recrystallized grains have a certain degree of growth. When the alloy is annealed at 620 °C for 1 h, the subgrains merge. The merging of subgrains causes the orientation of two or more subgrains to be consistent, which can be used as the nucleus of recrystallization and promote the occurrence of the recrystallization phenomenon. Also, the recrystallized grains of both groups of alloys increase.

From Figure 6, it can be seen that the softening phenomenon is not obvious when the three groups of Cu-Fe-Mg-Ti alloys are annealed in the temperature interval of 460 °C to 500 °C. From the TEM diagram in Figure 9, it can be seen that after 400 °C × 1 h heat preservation treatment, the alloy undergoes a recrystallization phenomenon, which indicates that when the alloy is annealed in this temperature range, the alloy’s recovery and recrystallization is the reason for the reduction in its hardness, but due to the low degree of recrystallization, the hardness value of the alloy does not change much. When the three groups of alloys are annealed in the temperature range of 500 °C to 580 °C, the degree of alloy restitution and recrystallization is intensified, consuming some of the dislocations, and it can be observed from Figure 8 that the density of dislocations decreases, with a softening effect on the alloy, and the hardness begins to decrease. When the alloy is annealed in a temperature range of 580 °C to 660 °C, the degree of alloy recovery and recrystallization is further aggravated, the dislocation density decreases sharply, the internal dislocation density of the alloy further decreases, the recrystallized grains grow significantly, and the phenomenon of alloy recovery and recrystallization is further aggravated.

In summary, when the Cu-Fe-Mg-Ti alloys are annealed from 460 °C to 580 °C, the softening is caused by restitution and recrystallization, and when the alloys are annealed from 580 °C to 660 °C, the alloys soften as a result of the combined effect of the restitution and recrystallization phenomena and the coarsening of the particles in the precipitated phase. The recrystallization ratio of the FT21 alloy is lower than that of the FT12 alloy and the FT11 alloy, which indicates that the recrystallization ratio of the alloy decreases and the softening property of the alloy improves when the ratio of iron to titanium is 2:1. Therefore, the softening temperature of the FT21 alloy is higher than that of the FT12 and FT11 alloys, and the softening temperature of the Cu-Fe-Mg-Ti alloy can be effectively increased when the ratio of iron to titanium is 2:1.

## 4. Conclusions

(1) Among three groups of Cu-Fe-Mg-Ti alloys with different Fe/Ti ratios, the best overall performance of the alloy is achieved when the Fe/Ti ratio is 2:1, and a peak hardness of 102.1 HV and an electrical conductivity of 39.7% IACS are reached 2 h after aging at 550 °C. The peak hardness of the FT21 alloy is reached 1 h after 70% cold rolling and aging at 550 °C, at which time the peak hardness is 166.5 HV and the electrical conductivity is 64.1% IACS.

(2) The precipitation phase of the FT21 alloy is spherical, the spherical precipitation particles are Fe_2_Ti with a hexagonal crystal system, and there is a [120]_Fe2Ti_//[$\bar{1}10$]_Cu_ site-directional relationship between the Fe_2_Ti phase and the substrate. The Fe2Ti precipitation phase was also obtained in the FT11 and FT12 alloys, with a uniform distribution of the organization.

(3) Three groups of Cu-Fe-Mg-Ti alloys with different Fe/Ti ratios were studied. The alloys with a Fe/Ti ratio of 2:1 have the best resistance to softening temperature, with a softening temperature of 600 °C, while the FT11 alloy has a softening temperature of 560 °C and the FT12 alloy has a softening temperature of 580 °C.

(4) The annealing softening mechanism of the Cu-Fe-Mg-Ti alloy within different temperature ranges is different. The main softening mechanisms of the alloys at low temperatures are reversion and partial recrystallization nucleation, and at high temperatures, the main softening mechanisms are the growth of recrystallized grains and coarsening of precipitated phases. For the Cu-Fe-Mg-Ti alloys with different Fe/Ti ratios, the best resistance to softening temperature is at 600 °C when the Fe/Ti ratio is 2:1.

## Figures and Tables

**Table 1:** Measured compositions of Cu-Fe-Mg-Ti under investigation (wt.%).

Fe/Ti Atomic Ratio	Number	Fe	Ti	Co	Mg	Cu
1:2	FT12	0.34	0.59	/	0.09	Bal
1:1	FT11	0.45	0.55	/	0.08	Bal
2:1	FT21	0.69	0.31	/	0.11	Bal

**Table 2:** Recrystallization degree of the FT12 alloy (%).

Temperature	FT12
500 °C	2.8
620 °C	28.6

## Data Availability

The original contributions presented in this study are included in the article. Further inquiries can be directed to the corresponding author.
